# Editorial: Special Issue “Implementation of Sensors and Artificial Intelligence for Environmental Hazards Assessment in Urban, Agriculture and Forestry Systems”

**DOI:** 10.3390/s21196383

**Published:** 2021-09-24

**Authors:** Sigfredo Fuentes, Eden Jane Tongson

**Affiliations:** Digital Agriculture, Food and Wine Research Group, Faculty of Veterinary and Agricultural Sciences, School of Agriculture and Food, The University of Melbourne, Parkville, VIC 3010, Australia; eden.tongson@unimelb.edu.au

Artificial intelligence (AI), together with robotics, sensors, sensor networks, internet of things (IoT) and machine/deep learning modeling, has reached the forefront towards the goal of increased efficiency in a multitude of application and purpose. The development and application of AI requires specific considerations, approaches, and methodologies. This special issue focused on the applications of AI to environmental systems related to hazard assessment in Urban, Agriculture and Forestry. A total of ten papers were published in this special issue, with topics ranging from reviewing the current climate-smart agriculture approaches for smart village development [1] to the integration of visible and infrared thermal cameras for automated urban green infrastructure monitoring on top of moving vehicles [2]; the implementation of machine learning to classify contaminant sources for urban water networks [3]; water network contamination assessment using machine learning in the UK [4]; future landscape changes, seismic and hazard assessment tested in Tabriz, Iran assessed using satellite remote sensing [5]; AI applied to a robotic dairy farm to assess milk productivity and quality traits using meteorological and cow data [6]; AI and computer vision from visible and infrared thermal images to obtain non-invasive biometrics from sheep to assess welfare [7]; the assessment of smoke contamination and smoke taint in wines due to bushfires using a low-cost electronic nose and AI [8]; the classification of smoke contaminated grapevine berries and leaves using chemical fingerprinting and machine learning [9]; and the detection of aphid infestation in wheat plants and insect-plant physiological interactions using low-cost electronic noses, chemical fingerprinting and machine learing [10].

The development of smart villages in Europe requires a framework to secure sustainability based on climate-smart agriculture. As argued by Adesipo et al. [1], these considerations need to be based on advances in technology to increase yield and minimize the farming losses associated with biotic and abiotic stresses. This approach will help for the efficient planning and management of smart villages with smart agriculture. The proposed frameworks will secure the success of these smart-agriculture practices under current and future climate change scenarios, making the system flexible and reactive based on recent smart technological advances related to sensor technologies for automated monitoring, data processing and reporting. Digital technological advances were reported for an automated urban green infrastructure monitoring using integrated visible and infrared thermal cameras in Fuentes et al. [2]. Studied in Melbourne, Australia, this system is a novel assessment method which utilizes moving vehicles as monitoring robots to assess tree by tree growth and water status using computer vision algorithms. It was suggested that this system could be used on public transport to support the city council’s management, maintenance and improvement of green infrastructure and as a potential tool to increase urban resiliency to climate change, specifically against the urban heat island effect.

One of the detrimental effects of reduced green infrastructure is the contamination of waterways and water networks. The study by Lučin et al. [3] proposed a classification system based on machine learning models (Neural Network and Random Forest) to predict the number of contaminant injections in the Richmond water supply, UK. This study also proposed that the implementation of these algorithms can be used to run simulations to detect potential contamination risks and nodes with a high probability of contamination, making a management system more predictive than reactive with such vital urban resources. Similar work was conducted by Grbčić et al. [4] to locate contamination sources in water networks with a combination of Artificial Neural Network (ANN) to classify pollution sources. Other types of hazard assessments in urban systems were based on a case study in Tabriz, Iran, by Mohammadi et al. [5]. Using satellite remote sensing to extract land information made it possible to predict landscape changes due to seismic activity with high accuracy ranging from 94 to 96%. These technological advances can be extrapolated to other cities with similar risks.

For agricultural systems, novel digital technologies were applied for farm animal welfare assessment based on weather information and cow data to predict milk productivity and quality through supervised machine learning [6]. The models developed presented high accuracies for correlation models in the range of R = 0.86 and R = 0.87, respectively. The proposed AI system’s automation can be implemented in robotic and conventional dairy farms to respond more efficiently to climatic anomalies, such as cold stress or heat waves, to maintain animal welfare. Heat stress in animal transport has recently been a focus of public concern due to the high mortality of animals transported by sea passing; for example, the Persian Gulf with 50 °C. A high level of heat stress can result in serious health issues to animals and ultimately death. Digital and AI technologies based on integrated visible and infrared thermal cameras were proposed by Fuentes et al. [7] to assess the physiological parameters of sheep in heat stress environments. The proposed models showed high accuracies to monitor the heart rate, respiration rate and skin temperature of animals. These digital technologies could help farmers manage their livestock more efficiently through objective assessments of animal welfare.

Climate change effects include the increased incidence, number and severity of climatic anomalies such as heatwaves and bushfires. These climatic anomalies have a specific impact on viticulture and winemaking, specifically with bushfires producing smoke contamination on leaves and berries, which are later passed to the wine through the fermentation process. These have been investigated in two studies focused on implementing digital technologies and machine learning modeling using low-cost electronic noses [8] and near-infrared spectroscopy to assess the levels of smoke contamination in berries and smoke taint in wines [9]. The models demonstrated high accuracy, showing the good potential of these approaches as practical options for grape-growers. The application of these tools offers an accurate, cost-effective and objective assessment of smoke contamination and taint in wines for efficient management purposes.

Finally, low-cost electronic noses and near-infrared spectroscopy were also implemented to assess the infestation of insects in plants and the insect-plant interaction [10]. This study presented a novel way to sniff aphid infestation in wheat plants and estimate plant physiological parameters using machine learning modeling. Models developed resulted in the high accuracy of monitoring insect numbers, early infestation and physiological parameters such as photosynthesis, transpiration and stomatal conductance, which usually require expensive instrumentation for single leaf measurements. This research also proposed a deployment system using unmanned aerial vehicles (UAV) to increase the spatial and temporal monitoring scales for more efficient assessments.

## Data Availability

Data availability is specified for every published paper.

## References

[1] Adesipo A., Fadeyi O., Kuca K., Krejcar O., Maresova P., Selamat A., Adenola M. (2020). Smart and Climate-Smart Agricultural Trends as Core Aspects of Smart Village Functions. Sensors.

[2] Fuentes S., Tongson E., Gonzalez Viejo C. (2021). Urban Green Infrastructure Monitoring Using Remote Sensing from Integrated Visible and Thermal Infrared Cameras Mounted on a Moving Vehicle. Sensors.

[3] Lučin I., Grbčić L., Čarija Z., Kranjčević L. (2021). Machine-learning classification of a number of contaminant sources in an urban water network. Sensors.

[4] Grbčić L., Lučin I., Kranjčević L., Družeta S. (2020). A machine learning-based algorithm for water network contamination source localization. Sensors.

[5] Mohammadi A., Karimzadeh S., Valizadeh Kamran K., Matsuoka M. (2020). Extraction of land information, future landscape changes and seismic hazard assessment: A case study of Tabriz, Iran. Sensors.

[6] Fuentes S., Gonzalez Viejo C., Cullen B., Tongson E., Chauhan S.S., Dunshea F.R. (2020). Artificial Intelligence Applied to a Robotic Dairy Farm to Model Milk Productivity and Quality based on Cow Data and Daily Environmental Parameters. Sensors.

[7] Fuentes S., Gonzalez Viejo C., Chauhan S.S., Joy A., Tongson E., Dunshea F.R. (2020). Non-Invasive Sheep Biometrics Obtained by Computer Vision Algorithms and Machine Learning Modeling Using Integrated Visible/Infrared Thermal Cameras. Sensors.

[8] Fuentes S., Summerson V., Gonzalez Viejo C., Tongson E., Lipovetzky N., Wilkinson K.L., Szeto C., Unnithan R.R. (2020). Assessment of Smoke Contamination in Grapevine Berries and Taint in Wines Due to Bushfires Using a Low-Cost E-Nose and an Artificial Intelligence Approach. Sensors.

[9] Summerson V., Gonzalez Viejo C., Szeto C., Wilkinson K.L., Torrico D.D., Pang A., De Bei R., Fuentes S. (2020). Classification of smoke contaminated Cabernet Sauvignon berries and leaves based on chemical fingerprinting and machine learning algorithms. Sensors.

[10] Fuentes S., Tongson E., Unnithan R.R., Gonzalez Viejo C. (2021). Early Detection of Aphid Infestation and Insect-Plant Interaction Assessment in Wheat Using a Low-Cost Electronic Nose (E-Nose), Near-Infrared Spectroscopy and Machine Learning Modeling. Sensors.

